# Modelling Coupled Oscillations in the Notch, Wnt, and FGF Signaling Pathways during Somitogenesis: A Comprehensive Mathematical Model

**DOI:** 10.1155/2015/387409

**Published:** 2015-03-17

**Authors:** Hong-yan Wang, Yan-xin Huang, Li-hua Zheng, Yong-li Bao, Lu-guo Sun, Yin Wu, Chun-lei Yu, Zhen-bo Song, Ying Sun, Guan-nan Wang, Zhi-qiang Ma, Yu-xin Li

**Affiliations:** ^1^National Engineering Laboratory for Druggable Gene and Protein Screening, Northeast Normal University, Changchun 130024, China; ^2^School of Computer Science and Information Technology, Northeast Normal University, Changchun 130117, China; ^3^Research Center of Agriculture and Medicine Gene Engineering of Ministry of Education, Northeast Normal University, Changchun 130024, China

## Abstract

Somite formation in the early stage of vertebrate embryonic development is controlled by a complicated gene network named segmentation clock, which is defined by the periodic expression of genes related to the Notch, Wnt, and the fibroblast growth factor (FGF) pathways. Although in recent years some findings about crosstalk among the Notch, Wnt, and FGF pathways in somitogenesis have been reported, the investigation of their crosstalk mechanisms from a systematic point of view is still lacking. In this study, a more comprehensive mathematical model was proposed to simulate the dynamics of the Notch, Wnt, and FGF pathways in the segmentation clock. Simulations and bifurcation analyses of this model suggested that the concentration gradients of both Wnt, and FGF signals along the presomitic mesoderm (PSM) are corresponding to the whole process from start to stop of the segmentation clock. A number of highly sensitive parameters to the segmentation clock's oscillatory pattern were identified. By further bifurcation analyses for these sensitive parameters, and several complementary mechanisms in respect of the maintenance of the stable oscillation of the segmentation clock were revealed.

## 1. Introduction

In the early stage of vertebrate embryonic development, the body is organized in a series of functionally equivalent units, each comprising a vertebra, its associated muscles, peripheral nerves, and blood vessels called somite [1]. Somites are progressively pinched off in pairs from the anterior end of two rods of mesenchymal tissue called PSM which lie either side of the caudal neural tube at the posterior end of the embryo [2]. It is accepted that somite formation is controlled by a complicated gene network named segmentation clock. For nearly a decade it has been known that the Notch pathway, the Wnt pathway, and the FGF pathway are the major components of the segmentation clock [3]. These pathways regulate the oscillatory expressions of their target genes, which play major roles in controlling somite formation [4–6].

In recent years mathematical models have been proposed to reveal the mechanisms of the signaling pathways in the segmentation clock. The first mathematical model of the Notch pathway was developed by Lewis [7]. They modelled a simple oscillation of Her1/7 gene expression in the embryo of zebrafish by introducing a negative feedback loop formed by the inhibition of Her1/7 protein to the transcription of itself. Later, Terry et al. proposed an improved oscillation model of Her1/7 gene expression by containing explicit equations of the transport, transcription, and translation of each molecule instead of time delay [8]. Differently from previous two models, a more complex model for the Notch pathway was proposed by Agrawal et al. to investigate cell fate decisions during somitogenesis of zebrafish [9]. The model contained three important genes Notch1, Hes1, and RBP-j*κ*, which formed a complicated regulation network consisting of several positive and negative feedback loops. The first mathematical model of the Wnt pathway in the segmentation clock was developed by Jensen et al. [10]. They modelled oscillatory gene expression of the Wnt pathway by introducing a negative feedback loop centered on Axin2. Subsequently, Pedersen et al. proposed another model of the Wnt pathway during somitogenesis, which contained the negative feedback loop centered on Dkk1 rather than Axin2 [11]. In addition, some mathematical models for the multiple pathways were also proposed. In 2007, Rodríguez-González et al. proposed the first crosstalk model between the Notch and Wnt pathways [12]. They demonstrated antiphase oscillation of clock genes between these two pathways. In 2008, Santillán and Mackey proposed an extremely abstract mathematical model for the interaction of the Notch and Wnt pathways in the PSM of mice embryo [13]. In their model, the Notch and Wnt pathways were abstracted as two isolated nodes, which inhibit themselves and activate the other, without considering their signal transduction process. In the same year, Goldbeter and Pourquié proposed a theoretical model that considered more complex interactions among the Notch, Wnt, and FGF pathways [14]. This model provided a framework for analyzing the dynamics of the segmentation clock in terms of a network of oscillatory modules involving the Notch, Wnt, and FGF pathways. In 2009, Kazama et al. proposed a crosstalk model between the Wnt and Notch pathways that considered the inhibiting of NICD to *β*-catenin in addition to the binding of NICD to Dsh [15]. In 2012, Tiedemann et al. presented an enhanced gene regulatory network model for the growing PSM of mice by many virtual cells and integrated Wnt3a and FGF8 gradient formation, periodic gene expression, and Delta/Notch signaling [16]. In 2013, our research group also proposed a crosstalk model, which contains multiple levels of crosstalk between the Notch and Wnt pathways [17]. Our model elucidated the mechanisms of the Notch and Wnt target genes' oscillatory expression, and the crosstalk mechanisms between the Notch and Wnt pathways. Although the proposed models have addressed different aspects of the segmentation clock dynamic behavior and yielded important insights into this system, the investigation of crosstalk mechanisms among the Notch, Wnt, and FGF pathways in somitogenesis from a systematic point of view is still lacking. Additionally, all these proposed models mainly focused on the feedback loops in the single pathway but did not explore the important roles of crosstalk among the multiple pathways to the stable oscillation of the segmentation clock. This inspired us to do some research on these issues.

Based on our previous work, in this paper we proposed a more comprehensive mathematical model for the Notch, Wnt, and FGF pathways in the segmentation clock. The simulation results elucidated that the concentration gradients of both Wnt and FGF signals along the PSM are corresponding to the start and stop of the segmentation clock. In order to investigate the molecular mechanisms for maintaining the stable oscillation of the segmentation clock, we conducted a complete sensibility analysis for the model parameters. A group of pivotal parameters that are critical to the periodicity of the segmentation clock was obtained and the bifurcation points of these parameters were determined. Furthermore, several complementary mechanisms involving the maintenance of the stable oscillation of the segmentation clock were identified by model simulation and bifurcation analysis.

## 2. Materials and Methods

### 2.1. Model Description

**Figure 1:**
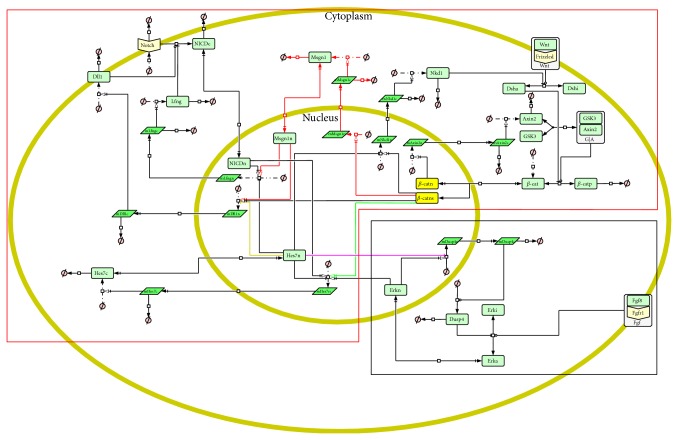
The schematic diagram of the model. The diagram is created using CellDesigner [18]. In the diagram, the outer yellow ellipse represents cytomembrane; the inner yellow ellipse represents nuclear membrane; the light green rectangles represent protein; the bottle green parallelograms represent mRNA; the white rectangles which contain light green rectangles represent complex; Φ represents the resultants of degradation reactions or reactants of combination reactions. The arrow lines represent biochemical reactions.

Based on our previous work [17] and the model of the FGF pathway proposed by Goldbeter and Pourquié [14], we established a more comprehensive crosstalk model for the Notch, Wnt, and FGF pathways in the segmentation clock. A schematic diagram of the model is shown in Figure 1. In this model we removed the RBP-j protein in the Notch pathway and the Lef1 protein in the Wnt pathway as well as all reactions associated with them for simplicity. Combination reactions of the NICD/Nkd1 proteins and the activated Dsh protein were replaced by new inhibition reactions. Other components, reactions, and most of their parameters in our previous model were left unchanged (in the red dotted frame in Figure 1). Subsequently, the FGF pathway model by Goldbeter and Pourquié (in the black dotted frame in Figure 1) and several levels of crosstalk among the Notch, Wnt, and FGF pathways were added to construct the new crosstalk model. According to the researches of Wittler et al. and Chalamalasetty et al., the Notch activator Mesogenin 1 (Msgn1) is directly induced by the Wnt signal so the Wnt pathway can regulate the Notch pathway through Msgn1 gene [19, 20]. A level of crosstalk between the Notch and Wnt pathways was thus established by Msgn1, and 8 new reactions were added to the model (see the red arrows in Figure 1). Another new level of crosstalk between the Notch and Wnt pathways is established by Hes7. The Hes7 gene is a target gene of the Notch pathway, at the same time it is an important repressor of the Notch pathway [21–25]. Recent finding of González et al. indicated that the expression of the Hes7 gene is also regulated by the *β*-catenin in the Wnt pathway [26]. So, induction of *β*-catenin (Wnt transcription factor) to the Hes7 gene was added to the new model (see the greenarrow in Figure 1). The research findings of Niwa et al. pointed out that the Hes7 is also activated by the FGF pathway, at the same time the FGF target gene Dusp4 is repressed by the Hes7 protein [25]. Thereby, the crosstalk between the Notch and FGF pathways was established by Hes7 in the model and 2 new reactions between the Notch and FGF pathways were added (see the blue and pink arrows in Figure 1). In addition, the researches of Jiang et al. indicated that DeltaC (homologous gene of Dll1) exhibits an oscillatory pattern and is repressed by Her7 (homologous gene of Hes7) in the zebrafish [27]. We added repression of Hes7 to Dll1 to the model (see the orange arrows in Figure 1). It is noteworthy that some Wnt target genes, such as Axin2, are expressed with an oscillatory pattern under the induction of *β*-catenin, while some other genes, such as Msgn1, are not. We guessed that the *β*-catenin protein regulates target genes with different kinetics. So, in this model, the *β*-catenin protein in the nucleus is divided into two groups (see the yellow rectangles in Figure 1).

### 2.2. Modelling Processes

Our model was established using ordinary differential equations (ODEs) (33 ODEs and 4 algebraic equations). The CellDesigner [18] was used to create the structure and the equations of the model, and then the file was exported from CellDesigner as SBML (Systems Biology Markup Language) format. The SBML file was imported into MATLAB and was translated to the format that the SBToolBox [28] can read. In the framework of the SBToolBox, we accomplished the tasks of parameter learning, parameter sensitivity analysis, and bifurcation analysis. All the simulations of the model were performed under the COPASI environment [29]. The methods used to determine the initial values and parameters as well as parameter sensitivity analysis can refer to our previous work [17]. The description to the signal transduction processes in the Notch, Wnt, and FGF pathways and the equations, parameters, and initial values of the model are presented at http://cs.nenu.edu.cn/Modelling/Support Materials.pdf. The model files established using CellDesigner, COPASI, and SBToolBox are presented at http://cs.nenu.edu.cn/Modelling/Model Files.rar.

### 2.3. The Approaches of Bifurcation Analysis

In this study, we mainly focused on the stable oscillation of the model. The oscillation pattern of target gene's expression has three statuses in respect of the period and amplitude of the oscillation: the stable oscillation, the unstable oscillation, and the nonoscillation. A bifurcation point of a parameter is defined as a value at which there is a sudden change in the oscillation pattern of target genes among the three statuses. We developed a fast method for bifurcation scanning, which divides search parameter space into a series of equidistant discrete points, where the distance can be set to 1/*n* of the range of the parameter (*n* = 400 in our research). The result of bifurcation analysis can be represented by an intuitive 3D/2D diagram named bifurcation diagram. For the bifurcation analysis of extracellular signals, we took every target gene as the output of the system, respectively. The outputs are 3D bifurcation diagrams whose 3D coordinate axes represent the parameter, time, and the expression of the target gene, respectively. For the one- or two-parameter bifurcation analysis, we took the Hes7 gene as the output of the system because the Hes7 gene is synchronously regulated by the Notch, Wnt, and FGF signals, and at the same time it also regulates the expression of the target genes of these pathways. The output of one-parameter bifurcation analysis is 3D bifurcation diagrams the same as the analysis of extracellular signals. The output of two-parameter bifurcation analysis is 2D bifurcation diagrams whose two coordinate axes represent the two analyzed parameters and the colour of every point in the diagrams reflects a stable degree of the Hes7 gene's oscillation.

The stable degree of the oscillation is calculated by the reciprocal of the standard variance of several continuous periods as

(1) $$\begin{array}{c} \text{DS}=\frac{1}{\sqrt{\left(1/n\right)\sum_{i=1}^{n}{\left(p_{i}-\bar{p}\right)}^{2}}+\delta }, \end{array}$$

where DS is the stable degree; *n* is the number of continuous periods; *p*_*i*_ is the period length of the *i*th period; $\bar{p}$ is the mean of all continuous periods; *δ* is very small number to void the denominator for 0 and here its value is 0.01.

## 3. Results and Discussions

### 3.1. Oscillation Simulation of the Model

**Figure 2:**
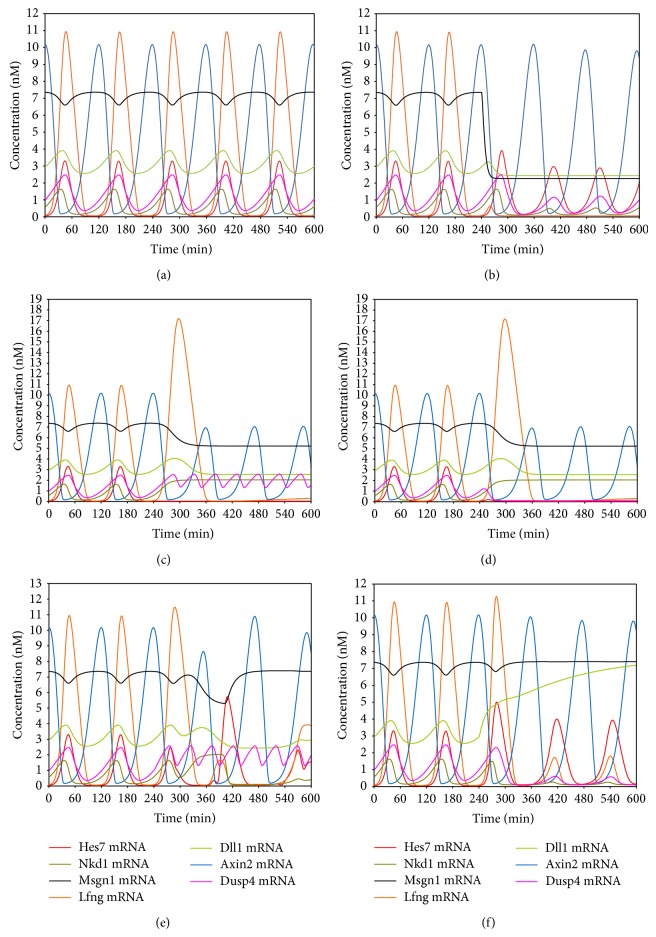
The simulation results of the model. (a) The oscillatory expression of the target genes under the condition of a constant extracellular Wnt and FGF signal. (b) The expression patterns of the target genes after the induction of the Wnt signal to Msgn1 was removed at time point 240 minutes. (c) The expression patterns of the target genes after the induction of the Wnt signal to the Hes7 gene was removed at time point 240 minutes. (d) The expression patterns of the target genes after the FGF protein was removed at time point 240 minutes. (e) The expression patterns of the target genes after the repression of the Hes7 protein to the Dusp4 gene was removed at time point 240 minutes. (f) The expression patterns of the target genes after the repression of the Hes7 protein to the Dll1 gene was removed at time points 240 minutes.

Because most components, reactions, and their parameters in the new model are same as our previous model and the model of Goldbeter et al., here we focussed on the newly added parts of the model. We first simulated the expression of the target genes under constant Wnt and FGF concentration (Wnt: 5 nM and FGF: 2 nM). As shown in Figure 2(a), all target genes except Msgn1 are expressed with an oscillatory pattern and their oscillatory period is about 120 minutes. Moreover, all the target genes except Axin2 oscillate synchronously, while the oscillation of the Axin2 gene is out of phase with others. We calculated the correlation of the expression levels of Lfng, Axin2, Dusp4, and Nkd1 genes to that of the Hes7 gene in the first five periods. The results show that Lfng, Dusp4, and Nkd1 are positive correlation with Hes7 and Axin2 is negative correlation with Hes7 in all periods (the results are not shown). Therefore, all the target genes reach a stable oscillation synchronously in phase or out of phase. These results are in good agreement with the results derived from wet experiment [25, 30–33]. Subsequently, we removed the induction of the Wnt signal to Msgn1 by setting *K*_*BsmMsgn*1_ (this parameter represents the dissociation constant between the *β*-catenin protein and the Msgn1 gene) to a very large value (100 nM) at time point 240 minutes. As shown in Figure 2(b), the Msgn1 gene is seriously downregulated immediately and the Dll1 and Lfng genes are also downregulated with Msgn1 and no longer oscillate, while other target genes that are not the direct targets of the Msgn1 gene are still expressed with oscillatory patterns. The simulation results indicate that the regulation of the Wnt pathway to the Notch pathway through Msgn1 is essential to the stable oscillations of Notch target genes. These are consistent with the findings of Wittler et al. and Chalamalasetty et al. [19, 20]. Similarly, we removed the induction of the Wnt signal to the Hes7 gene by setting *K*_*BsmHes*7_ (this parameter represents the dissociation constant between the *β*-catenin protein and the Hes7 gene) to a very large value (100 nM) at time point of 240 minutes. As shown in Figure 2(c), the Hes7 gene is not expressed immediately. This had been mentioned in the research of González et al. [26]. Lfng, Nkd1, and Dusp4 genes are upregulated and do not normally oscillate due to the disappearance of their repressor Hes7 [25, 32, 34]. The Wnt target gene Axin2 is still expressed with an oscillatory pattern but its expression level descends, and the expression level of Msgn1 descends too. This may be explained by the upregulation of the Wnt inhibitor Nkd1 [32]. So the induction of the Wnt signal to the Hes7 gene is essential to the normal expression of not only Hes7 but also many other genes in the segmentation clock because the Hes7 is an important repressor of many genes in the segmentation clock. Further, we removed the FGF8 protein at time point of 240 minutes which is the ligand of the FGF pathway. As shown in Figure 2(d), the Dusp4 and Hes7 genes are not expressed when the FGF8 is knocked out. This is consistent with the researches of Niwa et al. in 2007 [25]. Some Hes7 target genes, such as Lfng and Nkd1, are also not expressed as mentioned above (see Figure 2(c)), but normal Wnt signaling is maintained in the absence of FGF signaling. This is in accordance with the previous research results [25, 35]. We next removed the repression of the Hes7 protein to the Dusp4 gene by setting *K*_*HsmDusp*4_ (This parameter represents the dissociation constant between the Hes7 protein and the Dusp4 gene) to a very large value (100 nM) at time point of 240 minutes. As shown in Figure 2(e), Dusp4 is upregulated and shows an abnormal oscillatory pattern, which is in agreement with the research findings of Niwa et al. [25]. The Dusp4 protein is an inhibitor of the Hes7's regulator Erk [25, 36], and thus some Notch and FGF target genes also show abnormal oscillatory patterns due to the abnormal Hes7 gene caused by Dusp4. So the repression of the Hes7 gene to the Dusp4 gene is essential to the synchronous oscillation of not only the Dusp4 gene but also some Notch and FGF target genes. Lastly, we removed the repression of the Hes7 protein to the Dll1 gene by setting *K*_*HsmDll*1_ (this parameter represents the dissociation constant between the Hes7 protein and the Dll1 gene) to a very large value (100 nM) at time point of 240 minutes. As shown in Figure 2(f), the Dll1 is upregulated and does not oscillate. It further makes the expression level of the Hes7 gene ascend. As a result, all target genes of Hes7 are downregulated. So, the repression of the Hes7 protein to Dll1 is essential to its oscillatory expression. These results are in agreement with the experimental findings of Jiang et al. [27] and have been mentioned by the review of Pourquié in 2003 [37]. Above simulations demonstrated that our model can correctly simulate the expression of target genes in response to upstream signals in the segmentation clock. Removing newly added crosstalk levels in the model can result in the phenomena that agree well with the experimental results.

### 3.2. Influence of Extracellular Signals on the Stable Oscillation of the Model

**Figure 3:**
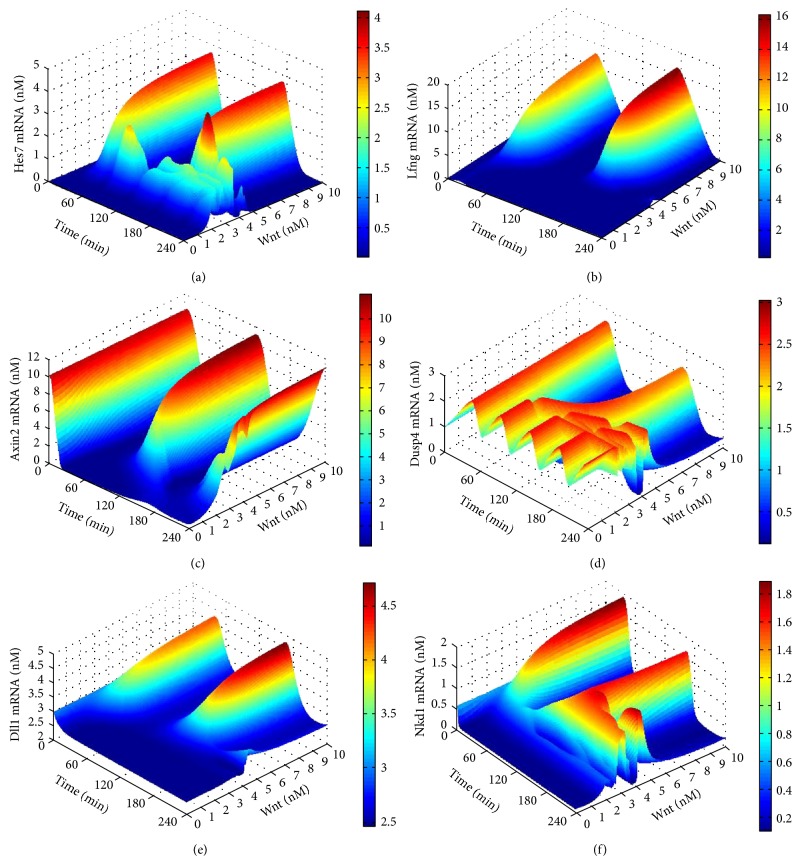
The results of the bifurcation scanning to the Wnt signal. The *X*-axis represents time. The *Y*-axis represents the concentration of the Wnt signal. The *Z*-axis and the colour bar both represent the concentration of the target genes' expression. (a) The expression level of the Hes7 gene in two periods during scanning the Wnt signals. (b) The expression level of the Lfng gene in two periods during scanning the Wnt signals. (c) The expression level of the Axin2 gene in two periods during scanning the Wnt signals. (d) The expression level of the Dusp4 gene in two periods during scanning the Wnt signals. (e) The expression level of the Dll1 gene in two periods during scanning the Wnt signals. (f) The expression level of the Nkd1 gene in two periods during scanning the Wnt signals.

Two extracellular signals, the Wnt signal and the FGF signal, are considered in our model. We first performed bifurcation scanning to the Wnt signal. The variable range of the Wnt signal was placed at 0 to 10 nM (5 nM is the normal concentration of the Wnt signal in the model) and the increasing step was set to 0.025. The expression levels of the target genes with Wnt signal changes in two periods were plotted in Figure 3. From the figure we can see that, when the Wnt signal is less than 2.2 nM, the Notch target genes Hes7 and Lfng, as well as all the Wnt target genes are not normally expressed, while the FGF target gene Dusp4 is highly expressed with an abnormal oscillatory pattern (refer to Figure 2(a)). We define this Wnt signal range as closed range. The low level of the Wnt signal causes the low expression of Hes7 gene, while the Hes7 is a suppressor of Dusp4, so its low expression brings about the abnormal oscillation of the Dusp4 [25]. When Wnt signal ranges from 2.2 nM to 4.5 nM, all target genes are expressed with abnormal oscillatory patterns. We define this Wnt signal range as transition range. When the Wnt signal is greater than 4.5 nM, all target genes reach stable oscillatory patterns; namely, their periods and amplitudes are no longer affected by the increase of the Wnt signal. We define this Wnt signal range as saturated range. Similarly, with the variable range from 0 to 4 nM and the increasing step of 0.01, we performed bifurcation scanning to the FGF signal, and defined its closed range, transition range, and saturated range as less than 0.24 nM, from 0.24 nM to 1.58 nM and greater than 1.58 nM, respectively. We finally performed a bifurcation scanning to Wnt-FGF two signals to investigate their dependence in respect to the stable oscillation of the segmentation clock. The results indicated that these two signals are independent and cannot substitute each other. The results of bifurcation scanning to the FGF signal and Wnt-FGF two signals can be seen in http://cs.nenu.edu.cn/Modelling/Support Materials.pdf.

The bifurcation scanning to the Wnt and FGF signals indicated that the lower levels of either the Wnt signal or the FGF signal prevents the segmentation clock from oscillation. There are respective saturated ranges for both Wnt and FGF signals in which perturbing the Wnt or FGF signal has no impact on the oscillatory characteristic of the segmentation clock. Although the two signals can both induce the expression of the target genes, they play roles independently without complementary relationship. Stable oscillation status of segmentation clock usually occurs in the posterior PSM at the beginning of a somite formation and is critical to somite formation. In the transition range, the segmentation clock graduates from stable oscillation to non-oscillation thereby showing an abnormal oscillation. This usually takes place in the anterior PSM before a somite formation is complete. In addition, when the Wnt or FGF signal is in the closed range target genes are not expressed. These results show that the concentration gradients of both Wnt and FGF signals along the PSM are corresponding to the whole process from start to stop of the segmentation clock. Based on above analysis results we can make the prediction that the somite size can be regulated by the concentration of either Wnt or FGF signal in the PSM through the model that may be experimentally tested. That is because either Wnt or FGF signal can control the clock's on-off and the closed range of the clock is just the boundary of new forming somite. In other words, when either Wnt or FGF signal in the PSM is increased, the closed range of the segmentation clock will move to the anterior of the PSM thereby causing the size of somite to become small. Reversely, when any signal is reduced, the closed range of the segmentation clock will move to the posterior thereby causing the size of somite to become large.

### 3.3. One-Parameter Bifurcation Analysis of the Oscillation Pattern of the Model

First of all, we performed a parameter sensitivity analysis to the model to recognize those important parameters to the stable oscillation of the system. The same as our previous study, the oscillatory period of the target genes of the model was taken as the output [17, 38, 39]. A parameter is defined important to the stable oscillation of the segmentation clock if its sensitivity value is greater than 1 or any target gene in the model loses its oscillation when this parameter is perturbed. We adopted six perturbation levels of parameters: low levels (±1%), medium levels (±5%) and high levels (±10%) in the parameter sensitivity analysis process.

The analysis results showed that the sensitive parameters can be divided into two groups: the one involved in the feedback loops of the pathways and the one relevant to the crosstalk between different pathways. We picked out these sensitive parameters and recorded them in Tables 1 and 2.

**Figure 4:**
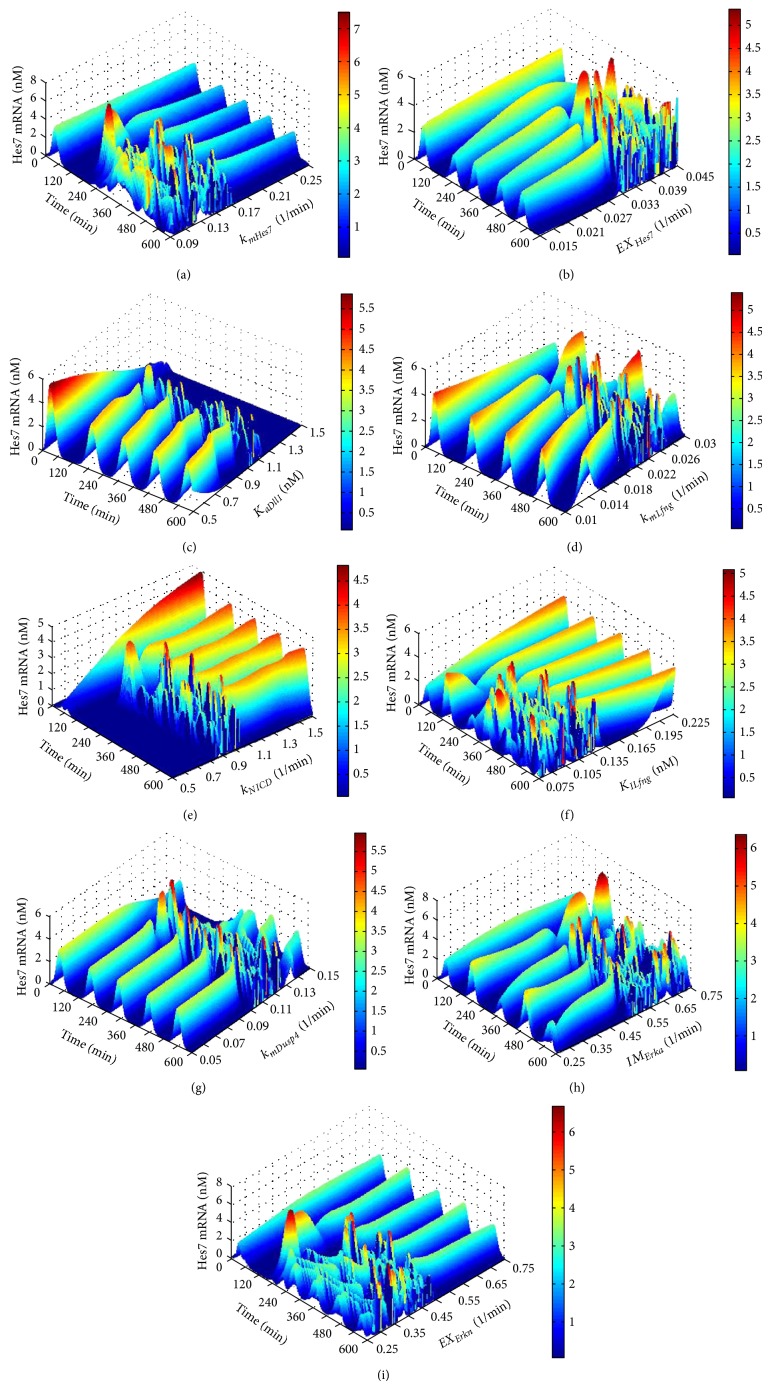
The results of one-parameter bifurcation analysis for the parameters in the feedback loops. The *X*-axis represents time. The *Y*-axis represents the values of the analyzed parameter. The *Z*-axis and the colour bar both represent the concentration of the target genes' expression. (a) The expression level of the Hes7 gene in respect to *k*_*mHes*7_ with the length of time 600 minutes. (b) The expression level of the Hes7 gene in respect to *EX*_*Hes*7_ with the length of time 600 minutes. (c) The expression level of the Hes7 gene in respect to *K*_*aDll*1_ with the length of time 600 minutes. (d) The expression level of the Hes7 gene in respect to *k*_*mLfng*_ with the length of time 600 minutes. (e) The expression level of the Hes7 gene in respect to *k*_*NICD*_ with the length of time 600 minutes. (f) The expression level of the Hes7 gene in respect to *K*_*ILfng*_ with the length of time 600 minutes. (g) The expression level of the Hes7 gene in respect to *k*_*mDusp*4_ with the length of time 600 minutes. (h) The expression level of the Hes7 gene in respect to *IM*_*Erka*_ with the length of time 600 minutes. (i) The expression level of the Hes7 gene in respect to *EX*_*Erkn*_ with the length of 600 minutes.

We next conducted a one-parameter bifurcation analysis for the sensitive parameters. The analysis results of the first group of sensitive parameters (refer to Table 1) are given in Figure 4. The sensitive parameters involved in the feedback loop centred Hes7 are *k*_*mHes*7_ (apparent first-order rate constant for synthesis of Hes7 protein) and *EX*_*Hes*7_ (apparent first-order rate constant for Hes7 protein exit from the nucleus). We can see from Figures 4(a) and 4(b) that when *k*_*mHes*7_ is greater than 0.173 min^−1^ and *EX*_*Hes*7_ is less than 0.0328 min^−1^ the Hes7 gene can reach a stable oscillation expression pattern. Besides this, it would lose its stable oscillation expression pattern. There are 4 sensitive parameters in the feedback loop centred on Lfng: *K*_*aDll*1_ (threshold constant for Notch cleavage into NICD by Dll1), *k*_*mLfng*_ (apparent first-order rate constant for synthesis of Lfng protein), *k*_*NICD*_ (apparent first-order rate constant for Notch cleavage into NICD) and *K*_*ILfng*_ (constant of inhibition by Lfng of Notch cleavage into NICD). When *K*_*aDll*1_ is less than 1.014 nM, *k*_*mLfng*_ is less than 0.021 min^−1^, *k*_*NICD*_ is greater than 1.01 min^−1^ and *K*_*ILfng*_ is greater than 0.137 nM, the Hes7 gene can reach a stable oscillation expression pattern. Apart from this, it would lose its stable oscillation expression pattern (see Figures 4(c) to 4(f)). The sensitive parameters involved in the feedback loop centred Dusp4 are *k*_*mDusp*4_ (apparent first-order rate constant for synthesis of Dusp4 protein), *IM*_*Erka*_ (apparent first-order rate constant for activated Erk entry into nucleus) and *EX*_*Erkn*_ (apparent first-order rate constant for activated Erk protein exit from the nucleus). When *k*_*mDusp*4_ is less than 0.102 min^−1^, *IM*_*Erka*_ is less than 0.517 min^−1^ and *EX*_*Erkn*_ is greater than 0.484 min^−1^, the Hes7 gene can reach a stable oscillation expression pattern. Besides this, it would lose its stable oscillation expression pattern (see Figures 4(g) to 4(i)). It is quite evident that there is only one bifurcation point for each of these parameters. Moreover, for some parameters, such as *EX*_*Hes*7_, *K*_*aDll*1_, *k*_*mLfng*_, *k*_*mDusp*4_, and *IM*_*Erka*_, when they are less than their bifurcation points, the segmentation clock can reach the stable oscillation, but for some other parameters, such as *k*_*mHes*7_, *k*_NICD_, *K*_ILfng_, and *EX*_*Erkn*_, the opposite is the case. This indicates that, for the stable oscillation of segmentation clock, some reactions, such as the exit of the Hes7 protein from the nucleus, the translation of the Lfng and Dusp4 genes, and the entry of the Erk protein into the nucleus, cannot be too fast, while some other reactions, such as the translation of the Hes7 gene, Notch cleavage into NICD and the exit of the Erk protein from the nucleus, must be fast enough. In addition, it was found that when the segmentation clock reaches the stable oscillation, changing the values of the parameters *K*_*aDll*1_, *k*_*mLfng*_, and *K*_*ILfng*_ can obviously change the oscillatory period of the segmentation clock. The feedback loop centred on Lfng in the Notch pathway is responsible for the regulation of the period of segmentation clock. This is in accord with the findings of Herrgen et al. [40].

**Figure 5:**
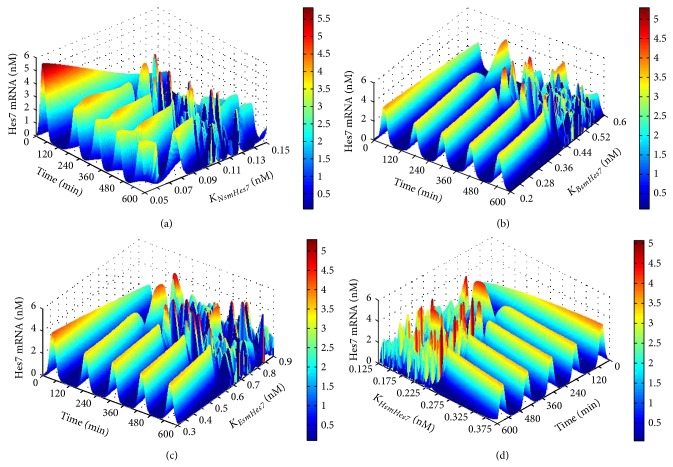
The results of one-parameter bifurcation analysis to the parameters in the reactions of the Hes7 gene's expression. The *X*-axis represents time. The *Y*-axis represents the values of the analyzed parameter. The *Z*-axis and the colour bar both represent the concentration of the target genes' expression. (a) The expression level of the Hes7 gene in respect to *K*_*NsmHes*7_ with the length of time 600 minutes. (b) The expression level of the Hes7 gene in respect to *K*_*BsmHes*7_ with the length of time 600 minutes. (c) The expression level of the Hes7 gene in respect to *K*_*EsmHes*7_ with the length of time 600 minutes. (d) The expression level of the Hes7 gene in respect to *K*_*HsmHes*7_ with the length of time 600 minutes.

The analysis results of the second group of sensitive parameters (refer to Table 2) can be seen in Figure 5 and in http://cs.nenu.edu.cn/Modelling/Support Materials.pdf. Taking the dissociation constants between the Hes7 gene and its transcription factors (*K*_*NsmHes*7_, *K*_*BsmHes*7_, *K*_*EsmHes*7_, and *K*_*HsmHes*7_) for example, from Figures 5(a) to 5(d), we can see that, when *K*_*NsmHes*7_ (dissociation constants between the Notch transcription factor NICD and the Hes7 gene) is less than 0.102 nM, *K*_*BsmHes*7_ (dissociation constants between the Wnt transcription factor *β*-catenin and the Hes7 gene) is less than 0.429 nM, *K*_*EsmHes*7_ (dissociation constants between the FGF transcription factor Erk and the Hes7 gene) is less than 0.626 nM and *K*_*HsmHes*7_ (dissociation constant between the repressor Hes7 protein and the Hes7 gene) is greater than 0.234 nM, the Hes7 gene can reach a stable oscillation expression pattern. Besides this, it would lose its stable oscillation expression pattern.

Therefore, these parameters have their respective one bifurcation points. Moreover, once the segmentation clock reaches the stable oscillation, changing the values of the parameter *K*_*NsmHes*7_ can obviously change the oscillatory period of the segmentation clock. The regulation of NICD to the Hes7 contributes to the regulation of the target genes' oscillatory period. Our analysis results also prove that the Notch pathway contributes to regulate the periods of the target genes [40]. All results of parameter sensitivity analysis of the model, including the other part of parameter sensitivity analysis in the second group, can be seen in http://cs.nenu.edu.cn/Modelling/Support Materials.pdf. The analysis results also show that for every sensitive parameter in the second group there is a bifurcation point which divides the parameter range into two parts. In one part, the segmentation clock can stably oscillate and in the other part not.

### 3.4. Two-Parameter Bifurcation Analysis of the Oscillation Pattern of the Model

**Figure 6:**
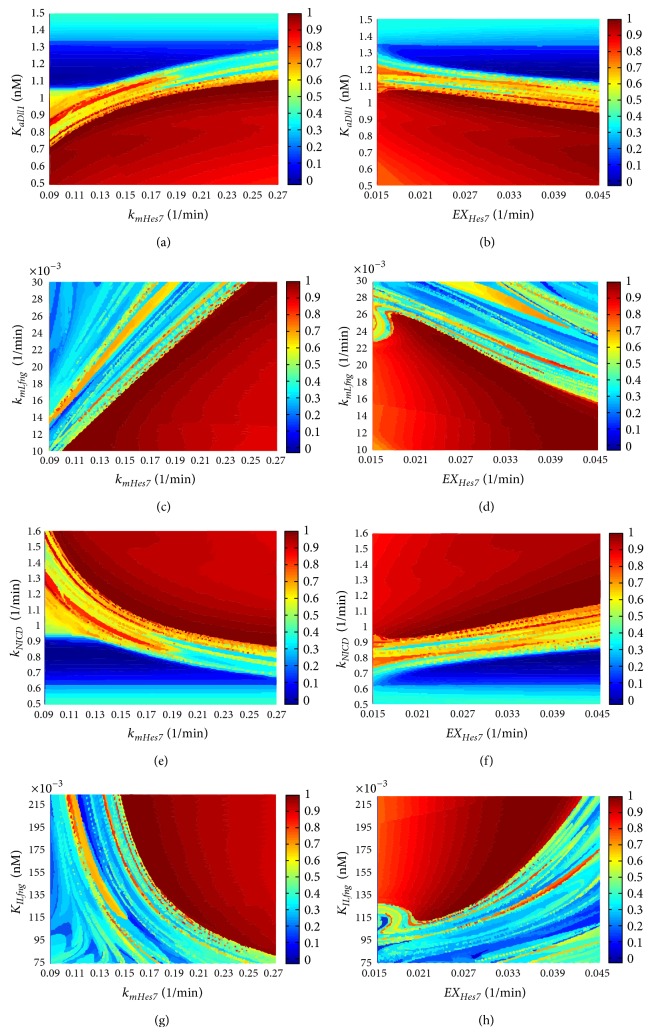
The results of two-parameter bifurcation analysis of the parameter pairs from the feedback loops centred on Hes7 and Lfng. The *X*-axis and *Y*-axis represent two parameters analyzed, respectively. The colour bar represents the normalized stable degree (see Equation (1)) of the Hes7 gene's oscillation at some a parameter value pair and it has no unit. (a) *k*_*mHes*7_-*K*_*aDll*1_. (b) *EX*_*Hes*7_-*K*_*aDll*1_. (c) *k*_*mHes*7_-*k*_*mLfng*_. (d) *EX*_*Hes*7_-*k*_*mLfng*_. (e) *k*_*mHes*7_-*k*_*NICD*_. (f) *EX*_*Hes*7_-*k*_*NICD*_. (g) *k*_*mHes*7_-*K*_*ILfng*_. (h) *EX*_*Hes*7_-*K*_*ILfng*_.

We further performed a two-parameter bifurcation analysis for the sensitive parameters by simultaneously perturbing a pair of parameters. We paired the parameters from different feedback loops in the first groups and from different levels of crosstalk among pathways in the second groups due to our focus on the relations between different feedback loops in pathways as well as the relations between the multi-levels of crosstalk among different pathways. The two-parameter bifurcation analysis results of the parameter pairs from the feedback loops centred on Hes7 and Lfng are shown in Figure 6. We can see that in every parameter plane there is a concentrated area at which the segmentation clock can stably oscillate and there is an obvious boundary between the stable oscillation area and the unstable oscillation area.

Moreover, for a pair of parameters associated with the two feedback loops, they are not independent of each other in respect of the oscillation of the segmentation clock. Changing one of them can obviously change the bifurcation point of the other one. That means there is a correlation between these two parameters. This correlation can be divided into two groups: positive correlation and negative correlation. In the positive correlation, with the increasing of one parameter value the bifurcation point value of the other parameter will increase. This situation exists among *k*_*mHes*7_-*K*_*aDll*1_ (see Figure 6(a)), *k*_*mHes*7_-*k*_*mLfng*_ (see Figure 6(c)), *EX*_*Hes*7_-*k*_*NICD*_ (see Figure 6(f)) and *EX*_*Hes*7_-*K*_*ILfng*_ (see Figure 6(h)). The negative correlation is opposite of the positive correlation. This situation exists among *EX*_*Hes*7_-*K*_*aDll*1_ (see Figure 6(b)), *EX*_*Hes*7_-*k*_*mLfng*_ (see Figure 6(d)), *k*_*mHes*7_-*k*_*NICD*_ (see Figure 6(e)) and *k*_*mHes*7_-*K*_*ILfng*_ (see Figure 6(g)). The analysis results for all the other parameter pairs can be seen in http://cs.nenu.edu.cn/Modelling/Support Materials.pdf. All these results indicated that if the segmentation clock loses the stable oscillation due to the change of certain a parameter, changing another parameter is able to rescue this loss of oscillation. All of these phenomena also exist between the parameters associated with other feedback loops as well as the parameters between multi-levels of crosstalk. That means there is a complementary mechanism between the parameters in different feedback loops as well as in different levels of crosstalk in the pathways.

**Figure 7:**
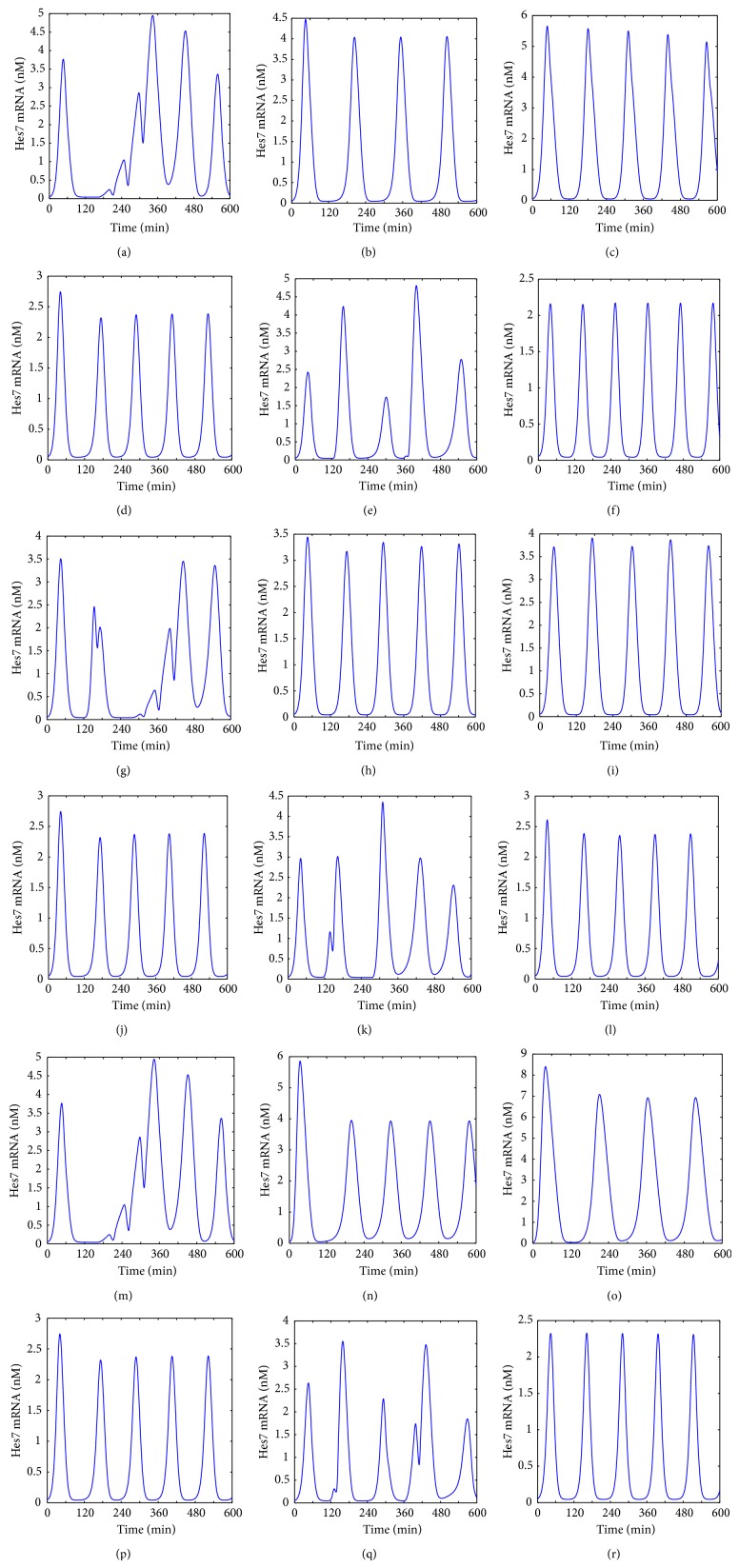
The expression of the Hes7 gene under the different conditions of *k*_*mHes*7_, *k*_*mLfng*_, *k*_*mDusp*4_ and *K*_*aDll*1_. (a) *k*_*mHes*7_ = 0.1 min^−1^. (b) *k*_*mLfng*_ = 0.01 min^−1^. (c) *k*_*mHes*7_ = 0.1 min^−1^ and *k*_*mLfng*_ = 0.01 min^−1^. (d) *k*_*mHes*7_ = 0.27 min^−1^. (e) *k*_*mLfng*_ = 0.03 min^−1^. (f) *k*_*mHes*7_ = 0.1 min^−1^ and *k*_*mLfng*_ = 0.03 min^−1^. (g) *k*_*mHes*7_ = 0.15 min^−1^. (h) *k*_*mDusp*4_ = 0.05 min^−1^. (i) *k*_*mHes*7_ = 0.15 min^−1^ and *k*_*mDusp*4_ = 0.05 min^−1^. (j) *k*_*mHes*7_ = 0.27 min^−1^. (k) *k*_*mDusp*4_ = 0.11 min^−1^. (l) *k*_*mHes*7_ = 0.27 min^−1^ and *k*_*mDusp*4_ = 0.11 min^−1^. (m) *k*_*mHes*7_ = 0.1 min^−1^. (n) *K*_*aDll*1_ = 0.5 nM. (o) *k*_*mHes*7_ = 0.1 min^−1^ and *K*_*aDll*1_ = 0.5 nM. (p) *k*_*mHes*7_ = 0.27 min^−1^. (q) *K*_*aDll*1_ = 1.1 nM. (r) *k*_*mHes*7_ = 0.27 min^−1^ and *K*_*aDll*1_ = 1.1 nM.

To further reveal these mechanisms we performed a series of simulation experiments to show the expression of the Hes7 gene under the condition of different parameter values. Taking 3 pairs of parameters from different feedback loops for example, (*k*_*mHes*7_-*k*_*mLfng*_, *k*_*mHes*7_-*k*_*mDsup*4_ and *k*_*mHes*7_-*K*_*aDll*1_), in Figure 7 we gave the experimental results. From Figure 7(a), we can see that when *k*_*mHes*7_ is set to 0.1 min^−1^ (the value of *k*_*mLfng*_ is 0.02 min^−1^ at this moment), the Hes7 gene shows an irregular oscillation. This is because the parameter value of *k*_*mHes*7_ is out of the oscillation range (see above section). When *k*_*mLfng*_ is set to 0.01 min^−1^ (the value of *k*_*mHes*7_ is 0.18 min^−1^ at this moment), the Hes7 gene shows a stable oscillation pattern but with a larger period (larger than 120 minutes, see Figure 7(b)). As noted above, *k*_*mLfng*_ contributes to regulate the period of target genes so reducing *k*_*mLfng*_ extends the period. When *k*_*mHes*7_ is set to 0.1 min^−1^ and *k*_*mLfng*_ is set to 0.01 min^−1^, the Hes7 gene shows a regular oscillation expression pattern with a period about 120 minutes (see Figure 7(c)). This indicated that reducing *k*_*mLfng*_ rescues the loss of the Hes7 gene's oscillation caused by reducing *k*_*mHes*7_. Conversely, when *k*_*mHes*7_ is set to 0.27 min^−1^ (the value of *k*_*mLfng*_ is 0.02 min^−1^ at this moment), the Hes7 gene shows an oscillation expression pattern with a period about 120 minutes (see Figure 7(d)). This is also apparent because the parameter value of *k*_*mHes*7_ is in the oscillation range and changing it almost does not affect the periods of target genes (see above section). But when *k*_*mLfng*_ is set to 0.03 min^−1^ (the value of *k*_*mHes*7_ is 0.18 min^−1^ at this moment), the Hes7 gene shows an irregular oscillation pattern (see Figure 7(e)). This happens because the value of *k*_*mLfng*_ is out of its oscillation range. When *k*_*mHes*7_ is set to 0.27 min^−1^ and *k*_*mLfng*_ is left unchanged at 0.03 min^−1^, the Hes7 gene shows a regular oscillation expression pattern although the period is a little shorter than the normal (see Figure 7(f)). This indicated that increasing *k*_*mHes*7_ rescued the loss of oscillation caused by increasing *k*_*mLfng*_.

This complementary relation also holds between *k*_*mHes*7_ and *k*_*mDusp*4_ (see Figures 7(g)–7(l)), between *k*_*mHes*7_ and *K*_*aDll*1_ (see Figures 7(m)–7(r)), and between the Hes7 gene and its four transcription factors (*K*_*HsmHes*7_-*K*_*NsmHes*7_, *K*_*HsmHes*7_-*K*_*BsmHes*7_, and *K*_*HsmHes*7_-*K*_*EsmHes*7_, the result can be seen in http://cs.nenu.edu.cn/Modelling/Support Materials.pdf). Because *k*_*mHes*7_, *k*_*mLfng*_, *k*_*mDusp*4_, and *K*_*aDll*1_ are in different feedback loops and *K*_*HsmHes*7_, *K*_*NsmHes*7_, *K*_*BsmHes*7_ and *K*_*EsmHes*7_ are in different levels of crosstalk, we proposed the hypothesis that this complementary relation from different feedback loops and from multi-levels of crosstalk in pathways enhance the antijamming ability of the segmentation clock and therefore contribute to maintain its stable oscillation.

From above results of the parameter bifurcation analysis we can predict that reducing the translation rate of the Hes7 protein through external stimuli in the PSM can block the stable oscillation of the segmentation clock. However, this blocking can be remedied by reducing the translation rate of the Lfng protein. Similarly, increasing the translation rate of the Hes7 protein through internal stimuli in the PSM can remedy the loss of the segmentation clock's stable oscillation due to the increasing of the translation rate of the Lfng protein. The same complementary relations also exist between the translation rate of the Hes7 protein and the translation rate of the Dusp4 protein. Of cause, all of these predictions need further verification through wet experiments.

## 4. Conclusions

In this study, we proposed a more comprehensive mathematical model to simulate the dynamics of the Notch, Wnt, and FGF pathways in the segmentation clock and focused on studying the molecular mechanisms for maintaining the stable oscillation of the segmentation clock through a series of simulation experiments. The simulation results show that, in the upstream of the segmentation clock, the concentration gradients of both Wnt and FGF signals along the PSM are corresponding to the whole process from start to stop of the segmentation clock. A group of important parameters that can significantly influence the oscillation of the segmentation clock were found and their stable ranges were determined. In addition, we found that when the oscillation of the segmentation clock is lost due to disturbing an important parameter, it can be rescued by altering another parameter. This complementary mechanism exists between different feedback loops and different levels of crosstalk in the pathways. We inferred that this complementary mechanism is one of the main causes of maintaining the stable oscillation of segmentation clock.

## Supplementary Material

The first Supplementary Material () contains all ODEs, parameters and initial values of the model . In addition, some simulation results that don't appear in the paper are also in it.
The second Supplementary Material () contains the model files created using CellDesigner, COPASI and SBToolBox of the MATLAB.

## Figures and Tables

**Table 1:** The sensitive parameters contained in the feedback loops.

Parameter perturbation	Parameters associated with the feedback loop centred on Hes7	Parameters associated with the feedback loop centred on Lfng	Parameters associated with the feedback loop centred on Dusp4
+1%			*k*_*mDusp*4_
			*IM*_*Erka*_

+5%		**K**_**aDll**1_	**k**_**mDusp**4_
	*EX*_*Hes*7_	k_mLfng_	**I** **M**_**Erka**_

+10%	*EX*_*Hes*7_	**K**_**aDll**1_	k_mDusp4_
		**k**_**mLfng**_	**I** **M**_**Erka**_

−1%		k_NICD_	EX_Erkn_

−5%	**k**_**mHes**7_	**k**_**NICD**_	**E** **X**_**Erkn**_
		K_ILfng_	

−10%	**k**_**mHes**7_	**k**_**NICD**_	**E** **X**_**Erkn**_
		**K**_**ILfng**_	

The thin font represents that the periods of the target genes are positive correlation to the parameters; the thin italics represent that the periods of the target genes are negative correlation to the parameters; the bold font represent that the target genes do not oscillate under that perturbation level.

**Table 2:** The sensitive parameters contained in the crosstalk among the pathways.

Parameter perturbation	Parameters associated with crosstalk between every two pathways					
+1%	V_MsmMsgn1_	V_MsmDll1_	K_EsmDusp4_	K_Hsmdll1_		
	*K*_*MsmDll*1_	*K*_*BsmDll*1_	*K*_*NsmHes*7_			
	**V**_**MsmDusp**4_	**K**_**BsmMsgn**1_				

+5%	*K*_*MsmDll*1_					
	**V**_**MsmLfng**_	**V**_**MsmNkd**1_	**V**_**MsmMsgn**1_	**V**_**MsmDusp**4_	**K**_**HsmLfng**_	**K**_**NsmHes**7_
	**K**_**BsmHes**7_	**K**_**EsmHes**7_	**K**_**BsmDll**1_	**K**_**HsmDusp**4_		

+10%	**V**_**MsmLfng**_	**V**_**MsmNkd**1_	**V**_**MsmMsgn**1_	**V**_**MsmDusp**4_	**K**_**HsmLfng**_	**K**_**NsmHes**7_
	**K**_**BsmHes**7_	**K**_**EsmHes**7_	**K**_**BsmDll**1_	**K**_**HsmDusp**4_		

−1%	V_MsmHes7_	V_MsmDll1_	K_BsmNkd1_	K_HsmDll1_	K_NsmLfng_	
	*V*_*MsmDusp*4_	*K*_*BsmDll*1_	*K*_*NsmHes*7_			
	**V**_**MsmMsgn**1_	**K**_**BsmMsgn**1_	**K**_**EsmDusp**4_			

−5%	**V**_**MsmHes**7_	**V**_**MsmDll**1_	**K**_**NsmLfng**_	**K**_**MsmLfng**_	**K**_**HsmHes**7_	**K**_**HsmDll**1_
	**K**_**BsmNkd**1_	**K**_**BsmMsgn**1_	**K**_**EsmDusp**4_			

−10%	**V**_**MsmHes**7_	**V**_**MsmDll**1_	**K**_**NsmLfng**_	**K**_**MsmLfng**_	**K**_**HsmHes**7_	**K**_**HsmDll**1_
	**K**_**BsmNkd**1_	**K**_**BsmMsgn**1_	**K**_**EsmDusp**4_			

The thin font represents that the periods of the target genes are positive correlation to the parameters; the thin italics represent that the periods of the target genes are negative correlation to the parameters; the bold font represent that the target genes do not oscillate under that perturbation level.
